# Relationship between health checkups and cancer screenings of wives and health checkups of their husbands: A cross-sectional study in Japan

**DOI:** 10.1016/j.pmedr.2024.102701

**Published:** 2024-03-23

**Authors:** Taeko Watanabe, Takehiro Sugiyama, Tomoko Ito, Chitose Kawamura, Jun Komiyama, Nanako Tamiya

**Affiliations:** a Department of Health Services Research, Institute of Medicine, University of Tsukuba, 1-1-1 Tennodai, Tsukuba, Ibaraki, Japan; b Health Services Research and Development Center, University of Tsukuba, 1-1-1 Tennodai, Tsukuba, Ibaraki, Japan; c Diabetes and Metabolism Information Center, Research Institute, National Center for Global Health and Medicine, 1-21-1 Toyama, Shinjuku, Tokyo, Japan; d Institute for Global Health Policy Research, Bureau of International Health Cooperation, National Center for Global Health and Medicine, 1-21-1 Toyama, Shinjuku, Tokyo, Japan; e Department of Health Services Research, Graduate School of Comprehensive Human Sciences, University of Tsukuba, 1-1-1 Tennodai, Tsukuba, Ibaraki, Japan

**Keywords:** Family, Married persons, Spouses, Middle aged, Early detection, Cancer, Early diagnosis, Japan

## Abstract

**Objectives:**

This study investigated the relationship between health checkups, cervical cancer screenings, and breast cancer screenings (collectively referred to as wellness examinations) of wives and health checkups of their husbands. We aimed to develop strategies to encourage wellness examinations among married individuals in Japan.

**Methods:**

This study used the 2019 Comprehensive Survey of Living Conditions, focusing on married couples aged 40–64. We analyzed the percentage of wives undergoing wellness examinations, grouped based on whether their husbands had undergone health checkups. Subsequently, multivariable modified Poisson regression analysis was performed considering sociodemographic and health-related factors. All analyses considered medical insurance of wives because wellness examination methods varied depending on medical insurance type.

**Results:**

The sample comprised 40,560 couples undergoing health checkups, 39,870 undergoing cervical cancer screening, and 39,895 undergoing breast cancer screening. Regardless of the medical insurance type of the wife, a significant positive association was observed between the wellness examination of wives and the health checkup of husbands across all age groups. After adjusting for covariates, prevalence ratios (95% confidence intervals) for wives whose husbands underwent health checkups were 2.24 (2.09–2.40) for national health insurance, 1.18 (1.16–1.21) for employee insurance (employee), and 1.53 (1.44–1.63) for employee insurance (family) for health checkups. Similar trends were observed in cervical and breast cancer screening.

**Conclusions:**

Wellness examinations of wives were associated with those of their husbands, suggesting that couples often share similar health-seeking behaviors. Hence, targeted interventions are important for couples who do not undergo wellness examinations.

## 1 Introduction

There is a global increase in the prevalence of noncommunicable diseases, including cardiovascular diseases, cancer, and lifestyle-related conditions (Organization, 2023, Abbafati et al., 2020). Consequently, there is an increasing need for preventive interventions, early detection strategies, and measures for preventing disease progression. Health checkups and cancer screenings, especially cervical cancer screenings and breast cancer screenings for women, have been recognized as valuable approaches to address these needs, as they lead to lower medical costs, early treatment, and favorable outcomes (Yoon et al., 2020, Liss et al., 2021, Kerlikowske et al., 1995, Magnus et al., 2011, Lawson et al., 1998, Vicus et al., 2015). Under the “Industrial Safety and Health Act in Japan,” employers are obligated to offer annual health checkups to employees. Similarly, the “Act on Assurance of Medical Care for Elderly People” mandates that insurance providers conduct annual health checkups for insured individuals aged between 40 and 74 years (Co-operation OfE, Development, 2019). Additionally, based on the “Health Promotion Act,” most local governments recommend women aged 20 years and above to undergo cervical cancer screenings every 2 years and women aged 40 years and above to undergo breast cancer screenings every 2 years (Co-operation OfE, Development, 2019, Hospital, 2023). However, there is a disparity between the targeted rates stipulated by the government for health checkups and cancer screenings (health checkups: at least 70 % by 2017; cervical cancer screening and breast cancer screening: 50 % by 2022) and the actual rates (health checkups: 55.6 % in 2019; cervical cancer screening: 43.7 % in 2019; and breast cancer screening: 47.4 % in 2019) (Nutrition NIoHa, 2023). These results underscore the need for effective recommendations and interventions to bridge this gap and achieve the targeted rates.

Marriage is considered as a factor that influences various aspects of the lives, including health behaviors, of individuals. Studies have consistently revealed a remarkable concordance in health-related habits and practices among married partners (Meyler et al., 2007, Di Castelnuovo et al., 2009). Several hypotheses have been proposed to explain the underlying reasons for this concordance. The first hypothesis known as “*assortative mating*” suggests that individuals with similar traits are inclined to marry each other. The second hypothesis, the “*shared resource hypothesis*,” proposes that couples tend to share common environmental and economic resources. Another aspect is the presence of “*social control*,” where one partner exercises influence over the other. Finally, there is the phenomenon of “*mood convergence*” or “*affective contagion*,” suggesting that emotions can be influenced by the other partner (Meyler et al., 2007). Although specific mechanisms may vary across societies and couples, it is important to recognize the close relationship between spousal and personal health behaviors.

There is limited research regarding the concordance in health checkups and cancer screenings among married partners. This study aimed to investigate the relationship between engagement of wives in health checkups, cervical cancer screenings, and breast cancer screenings (collectively referred to as wellness examinations) and the participation of their husbands in health checkups. We hypothesized that health behaviors of married partners are associated, thereby certain couples undergo these screenings and certain do not. Our findings will provide valuable insights for developing effective strategies to promote wellness examinations among married individuals by understanding the consistency in the behavior of married couples.

## 2 Methods

### 2.1 Data

We performed a cross-sectional study using data from the 'household cards' and 'health cards' of the 2019 Comprehensive Survey of Living Conditions (CSLC)14. The CSLC is an official statistics survey conducted by the Ministry of Health, Labour, and Welfare (MHLW). It collects nationally representative information regarding households and their members and covers basic living conditions, such as family structure, health status, use of health and long-term care services, income, and savings. The sampling method was based on the enumeration districts (EDs) defined in the census, each of which included approximately 50 households. For the 2019 CSLC, 5,530 EDs were randomly sampled from the EDs in the 2015 census. All households (approximately 300,000) and members (approximately 720,000) in the selected EDs were surveyed using self-administered questionnaires.

In this study, we focused on couples in which both spouses were aged between 40 and 64 years. A married couple was defined as a male head of the household and a female spouse or a female head of the household and a male spouse living together. This age range was chosen because it aligns with the general working age in Japan and corresponds to the age group in which health and wellness examinations are institutionally offered. As regulated in Japan, there were no households with multiple household heads or spouses, and couples were identified one to one.

**Fig. 1:**
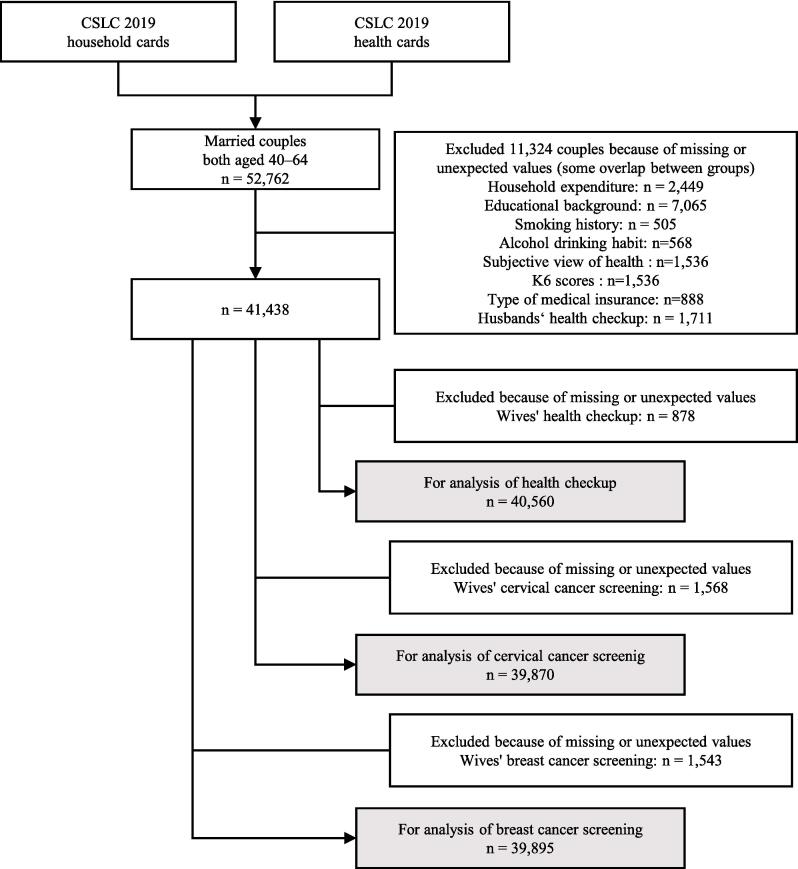
Flow chart describing the sampling process for the cross-sectional study on the relationship between health checkups and cancer screenings among wives, and their husbands' health checkups, using a nationally representative database from 2019 in Japan.

To ensure data quality, we excluded couples with missing values for the variables used in the analysis. A flowchart of the sampling procedure is presented in Fig. 1, illustrating the selection process of the study sample.

### 2.2 Measurements

The outcome variable of interest was whether wives had undergone wellness examinations within the recommended timeframe (i.e., health checkups within the past year, cervical cancer screenings within the past two years, and breast cancer screening within the past two years). This information was assessed based on their responses to the question, “Have you had a health checkup in the past year?” and “Have you undergone cervical cancer screening or breast cancer screening in the past two years?“.

The primary exposure variable of interest was whether the husband had undergone a health checkup within the past year. The criterion for defining the husband as having undergone a health checkup in the past year was the same as that used for the outcome variable.

Covariate information included the place of residence of the couple, household expenditure, presence of preschool children in the household, characteristics of the wife (age, educational background, smoking history, alcohol consumption habits, and subjective view of health, and K6 scores). Previous studies indicate that these factors are associated with the likelihood of undergoing wellness examinations (Iwasaki et al., 2003, Funahashi, 2013, Noguchi and Shen, 2019, Otsuka et al., 2021, Kawamura et al., 2023). Place of residence was categorized into three groups: large cities, medium or small cities, and rural areas. Household expenditure was represented by the logarithm of total household expenditure in month of May in the survey year. Preschool children in the household were categorized as present or absent based on the presence of children under 6 years old in the household. Age was divided into three groups: 40–49 years, 50–59 years, and 60–64 years. Educational background was classified into attendance at a college, university or graduate school, or not. Smoking history was categorized as never smoked, current smoker, or former smoker. Drinking habits were categorized based on whether the participants consumed more than 180 mL of sake on average per day. Subjective views on health were categorized as “good or normal” and “not good.” K6 scores were divided into two groups: less than 4 points and 5 points or more.

### 2.3 Statistical analyses

First, we calculated the proportion of wives who were undergoing wellness examinations. Then, we classified them based on age group and whether their husbands were undergoing health checkups, and determined the degree of concordance within the couples using the χ2 test.

Second, we performed a multivariable modified Poisson regression analysis. The primary outcome variable of interest was the utilization of wellness examinations by wives while the main exposure variable was the use of health checkups by husbands. The analysis included the following covariates as independent variables: place of residence; household expenditure; preschool children in the household; characteristics of the wife, such as age, educational background, smoking history, alcohol consumption habits, and subjective view of health; and K6 scores. For the sensitivity analysis, we performed multiple imputations by chained equations (MICE) using all variables, including outcomes. We generated five complete filled-in datasets and replaced each missing value with a set of plausible substitutes. Additionally, we conducted a supplementary analysis by reversing the exposure and outcome, with wives' health checkups as the exposure and husbands' health checkups as the outcome, with the characteristic of wives as covariates. Furthermore, we conducted a supplementary analysis by including additional covariates of characteristics of the husband, such as age, educational background, smoking history, alcohol consumption habits, and subjective view of health; and K6 scores.

All analyses were stratified based on the type of medical insurance of the wife, which included national health insurance, employee insurance (employee), and employee insurance (family). In the target age group of this study, the Japanese health insurance system mandates coverage based on employment status. Employees who have reached a certain employment threshold are enrolled in employee insurance (employee), dependents of those who meet the threshold are covered by employee insurance (family), while other individuals, including the self-employed and the unemployed, are included under national health insurance (Organization, 2023). This stratification is essential because the method of obtaining wellness examinations in Japan varies depending on the type of medical insurance.

All statistical analyses were performed using the Stata software (version 17.0; Stata Corp LP, College Station, TX, USA). *p* < 0.05 was considered statistically significant. This study adhered to the STROBE (Strengthening the Reporting of Observational Studies in Epidemiology) guidelines for the study design and manuscript preparation to ensure methodological rigor and transparent reporting of the results.

### 2.4 Ethical approval

Ethical approval was obtained from the Ethics Committee of the University of Tsukuba, Ibaraki, Japan (approval number 1664–2). This study used anonymized data obtained from the MHLW, and the need for informed consent was waived.

## 3 Results

In total, 40,560 married couples were included in the health checkup analysis, 39,870 married couples in the cervical cancer screening analysis, and 39,895 married couples in the breast cancer screening analysis. These couples belonged to the age group of 40–64 years and had no missing data. Detailed characteristics of the participants are presented in Table 1.

**Table 1:** Characteristics of couples participating in the cross-sectional study on the relationship between health checkups and cancer screenings among wives, and their husbands' health checkups, using a nationally representative database from 2019 in Japan.

Characteristics		Health checkup analysis		Cervical cancer analysis		Breast cancer analysis	
		(n = 40.560)		(n = 39,870)		(n = 39,895)	
		n	(%)	n	(%)	n	(%)
**Characteristics of couples**							
Residential areas	Large cities	8,717	(21.5 %)	8,553	(21.5 %)	8,559	(21.5 %)
	Medium or small cities	27,298	(67.3 %)	26,838	(67.3 %)	26,852	(67.3 %)
	Rural areas	4,545	(11.2 %)	4,479	(11.2 %)	4,484	(11.2 %)
Total household expenditures (ten thousand yen/month)	mean ± SD	29.29 ± 28.53		29.26 ± 28.18		29.27 ± 28.20	
Preschool children in the household	Presence	37,968	(93.6 %)	37,331	(93.6 %)	37,356	(93.6 %)
	Absence	2,592	(6.4 %)	2,539	(6.4 %)	2,539	(6.4 %)

**Characteristics of husbands**							
Receiving health check-up	Presence	35,390	(87.3 %)	34,807	(87.3 %)	34,831	(87.3 %)
	Absence	5,170	(12.7 %)	5,063	(12.7 %)	5,064	(12.7 %)

**Characteristics of wives**							
Receiving wellness examination	Presence	30,127	(74.3 %)	17,895	(44.9 %)	17,336	(43.5 %)
	Absence	10,433	(25.7 %)	21,975	(55.1 %)	22,559	(56.5 %)
Age group	40–49	17,880	(44.1 %)	17,560	(44.0 %)	17,569	(44.0 %)
	50–59	17,637	(43.5 %)	17,349	(43.5 %)	17,335	(43.5 %)
	60–64	5,043	(12.4 %)	4,961	(12.4 %)	4,971	(12.5 %)
Educational background	Colleges, universities or graduate schools	33,564	(82.8 %)	33,004	(82.8 %)	33,028	(82.8 %)
	Other	6,996	(17.2 %)	6,866	(17.2 %)	6,867	(17.2 %)
Smoking historiy	Never smoked	35,660	(87.9 %)	35,002	(87.8 %)	35,024	(87.8 %)
	Current smokers	3,773	(9.3 %)	3,692	(9.3 %)	3,694	(9.3 %)
	Former smokers	1,187	(2.9 %)	1,176	(2.9 %)	1,177	(3.0 %)
Alcohol drinking habit	Drinking more than 180 mL on average per day	36,961	(91.1 %)	36,345	(91.2 %)	36,366	(91.2 %)
	Other	3,599	(8.9 %)	3,525	(8.8 %)	3,529	(8.8 %)
Subjective views of health	Good or normal	36,172	(89.2 %)	35,554	(89.2 %)	35,578	(89.2 %)
	Not good	4,388	(10.8 %)	4,316	(10.8 %)	4,317	(10.8 %)
K6	< 4	28,060	(69.2 %)	27,550	(69.1 %)	27,572	(69.1 %)
	>= 5	12,500	(30.8 %)	12,320	(30.9 %)	12,323	(30.9 %)
Medical insurance	National insurance	5,082	(12.5 %)	4,983	(12.5 %)	4,989	(12.5 %)
	Employee insurance(employee)	16,320	(40.2 %)	16,006	(40.1 %)	16,011	(40.1 %)
	Employee insurance(family)	19,158	(47.2 %)	18,881	(47.4 %)	18,895	(47.4 %)

**Fig. 2:**
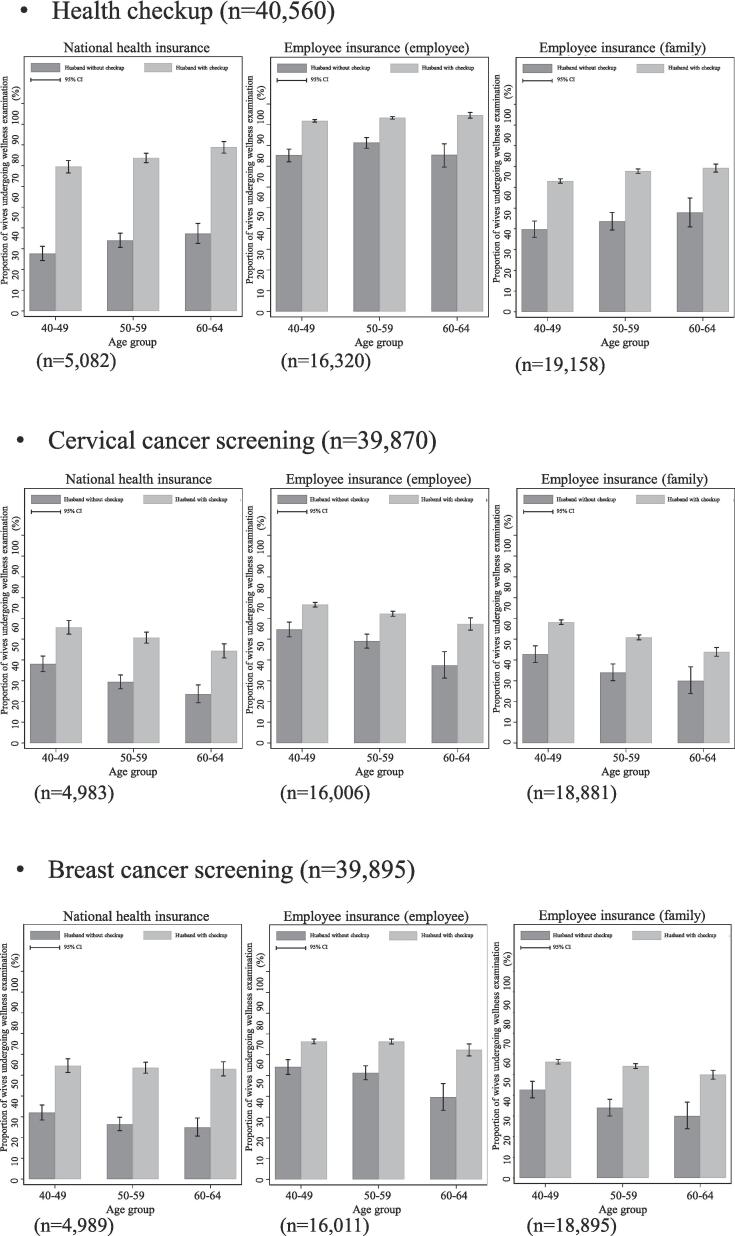
Proportion of wives undergoing wellness examination classified according to whether their husbands were undergoing health checkup based on stratified age group, using a nationally representative database from 2019 in Japan. *Note:* (1) Stratification was based on the wife's medical insurance type, including national health insurance, employee insurance (employee), and employee insurance (family); (2) Statistical differences were observed in the proportion of wives undergoing wellness examinations based on whether their husbands were undergoing health checkups across all age groups of all the type of medical insurance of the wife (the χ2 test, P < 0.05).

Regardless of the type of medical insurance of the wife, a significant positive association was observed between the wellness examination of wives and the health checkup of their husbands across all age groups, as shown in Fig. 2.

**Fig. 3:**
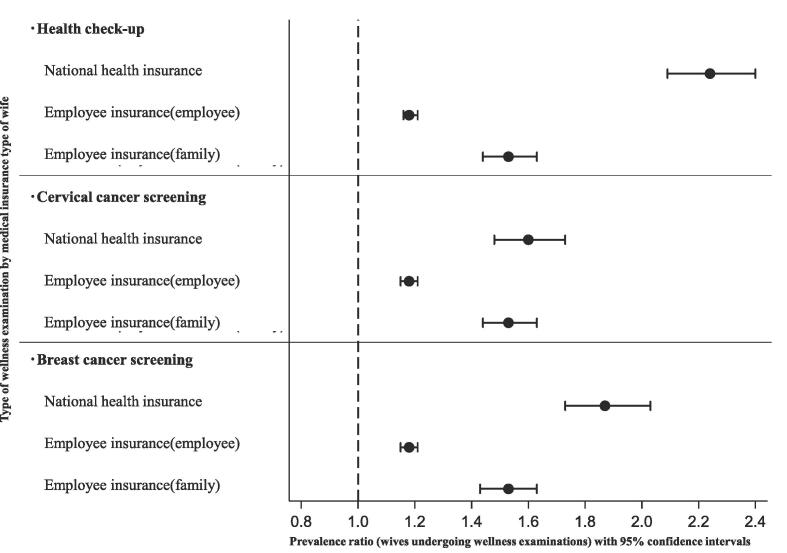
Prevalence ratios (95% confidence intervals) for undergoing wellness examinations among wives whose husbands underwent health checkups compared to wives whose husbands did not, using a nationally representative database from 2019 in Japan. *Note:* (1) Stratification was based on the wife's medical insurance type, including national health insurance, employee insurance (employee), and employee insurance (family); (2) Adjusted for place of residence; household expenditure; preschool children in the household; wife's status namely, age, education, smoking history, drinking habits, and subjective health perceptions; and K6 scores.

In the modified Poisson regression analysis, after adjusting for various factors related to health checkups, the prevalence ratio (95 % confidence interval) for wives whose husbands had undergone health checkups as compared with wives whose husbands had not undergone health checkups was as follows: 2.24 (2.09–2.40) for national health insurance, 1.18 (1.16–1.21) for employee insurance (employee), and 1.53 (1.44–1.63) for employee insurance (family). In terms of cervical cancer screening, the prevalence ratio (95 % confidence interval) for wives whose husbands underwent health checkups as compared with wives whose husbands did not undergo health checkups was 1.60 (1.48–1.73) for national health insurance, 1.18 (1.15–1.21) for employee insurance (employee), and 1.53 (1.44–1.63) for employee insurance (family). For breast cancer screening, the prevalence ratio (95 % confidence interval) for wives whose husbands underwent health checkups as compared with wives whose husbands did not undergo health checkups was 1.87 (1.73–2.03) for national health insurance, 1.18 (1.15–1.21) for employee insurance (employee), and 1.53 (1.43–1.63) for employee insurance (family) (Fig. 3). Details of the modified Poisson regression analysis results are presented in the Supplementary Table 1. Sensitivity analyses performed using MICE yielded similar results (Supplementary Fig. 1). Additionally, by conducting a supplementary analysis where the roles of exposure and outcome were inverted with wives' health checkups as the exposure and husbands' health checkups as the outcome yielded similar results (Supplementary Fig. 2). Incorporating additional covariates reflecting the husbands' characteristics into the analysis also produced similar results. (Supplementary Fig. 3).

## 4 Discussion

The non-adjusted analysis results (Fig. 2) revealed a significant positive association between wellness examinations of wives and health checkups of their husbands across all age groups, irrespective of the type of medical insurance. This suggests that couples tend to have similar health-seeking behaviors, with some couples prioritizing these wellness examinations together whereas others do not. While each individual's motivation for obtaining a wellness examination may vary, such as primary or secondary prevention, it is important that spouses behave similarly. The overall high participation rate among women with employee insurance (employee) can be attributed to the convenience of workplace health checkups, which may include cancer screenings (Co-operation OfE, Development, 2019). By contrast, wives with national health insurance or employee insurance (family) are responsible for arranging their own appointments, potentially leading to decreased participation rates. Even among wives covered by employee insurance (employees), the likelihood of undergoing wellness examinations correlated with whether their husbands also underwent these examinations. This highlights spousal concordance in wellness examinations, similar to other health behaviors (Macario and Sorensen, 1998, Graham and Braun, 1999, Jurj et al., 2006). Therefore, when recommending wellness examinations, insurers should consider the wellness examination status of the spouses and provide strong recommendations, if the spouse has not yet undergone these examinations.

The results of the multivariable modified Poisson regression analysis (Fig. 3), which was adjusted for various factors, further support the association between health checkups of husbands and wellness examinations among wives. The findings from sensitivity analyses employing MICE, along with the supplementary analysis that reversed the exposure and outcome or incorporating additional covariates reflecting the husbands' characteristics consistently supported the association shown in the main analysis. However, the impact of the health checkups of husbands on the likelihood of wives undergoing wellness examinations differs depending on the type of medical insurance. Specifically, the relationship between the wellness examinations of wives and the health checkups of husbands is relatively weak when the wife has employee insurance (employee). It can be considered that this is due to the strong influence of workplace recommendations.

To improve wellness examination rates among married individuals, it is crucial to develop intervention strategies that consider the spousal influence of couples while considering differences in medical insurance. First, based on the findings of this study, encouraging one partner in a married couple when both partners do not undergo wellness examinations may positively affect the other partner. If spouses do not undergo wellness examinations, actively promoting these by insurers for individuals with national health insurance or employee insurance (family) and leveraging workplace environments for those with employee insurance (employee) may be effective strategies. Moreover, based on the findings of this study, we hypothesize that concurrently targeting both spouses may have a greater probability of inducing behavioral modifications than concentrating only on a single spouse. These strategies may involve co-invitations for wellness examinations (Van Jaarsveld et al., 2006) and the implementation of couple-based educational programs (Lee et al., 2014). However, notably, changing the behavior of couples who do not receive wellness examinations may pose challenges, as suggested by a previous study (Manne et al., 2013).

To the best of our knowledge, this is the first study to find an association between wives' and husbands' wellness examinations in Japan, using a nationally representative database. Moreover, our results may help develop effective strategies to promote wellness examinations among married individuals. This study highlights the importance of understanding the association between wellness examinations of spouses of a married couple. Recognizing the influence of spousal behaviors and implementing targeted interventions may improve the rates of wellness examinations among married individuals. Previous studies in Japan (Watanabe et al., 2020) and worldwide (Hippisley-Cox et al., 2002, Leong et al., 2014, Wang et al., 2017) have demonstrated an increased likelihood of married couples developing shared non-communicable diseases. Therefore, it is crucial not to overlook couples in which both partners may not be in good health.

This study has some limitations. First, owing to its cross-sectional nature, causal relationships could not be established. Although various theories have been proposed to explain spousal concordance, determining the most influential factors remains challenging. Hence, a suitable cohort study should be performed to mitigate this limitation. Second, as this study relied on self-administered surveys, there were instances of missing data resulting from non-responses. To address this issue, we performed a sensitivity analysis using MICE and obtained similar results. However, we must acknowledge the possibility that missing data may have affected the results to a certain extent. In addition, due to data limitations, we were only able to identify the household head and their spouse as a couple. However, the number of couples omitted from the analysis is not significant given that Japan is moving toward nuclear families and that households in 2019 average 2.39 individuals (Ministry of Health LaW, 2021). Moreover, ideally, some variables such as the length of marriage, the quality of the relationship between the spouses, and health knowledge and awareness should have been included in the covariates. However, this was not possible due to a lack of data. Also, ideally, we would have excluded patients with cervical and breast cancer from the corresponding analyses. However, this was not feasible due to insufficient disease information.

## 5 Conclusions

This study revealed an association between wellness examinations of the husbands and their wives, thereby suggesting that couples often share similar health-seeking behaviors. This highlights the importance of targeted interventions for couples who do not undergo wellness examinations together. Future research should explore effective approaches to promote participation in wellness examinations among couples who do not undergo these examinations.

## Funding

This work was supported by a Grant-in-Aid for Challenging Research (Pioneering) [22K 18258] and a grant-in-aid from the Ministry of Health, Labour and Welfare; Health, Labour and Welfare Sciences Research Grant, Research on Region Medical [21IA1010].

## CRediT authorship contribution statement

**Taeko Watanabe:** Writing – review & editing, Writing – original draft, Methodology, Investigation, Conceptualization. **Takehiro Sugiyama:** Writing – review & editing, Writing – original draft, Methodology. **Tomoko Ito:** Writing – review & editing, Writing – original draft, Formal analysis. **Chitose Kawamura:** Writing – review & editing, Writing – original draft, Data curation. **Jun Komiyama:** Writing – review & editing, Writing – original draft, Data curation. **Nanako Tamiya:** Writing – review & editing, Writing – original draft, Supervision, Methodology.

## Declaration of competing interest

The authors declare that they have no known competing financial interests or personal relationships that could have appeared to influence the work reported in this paper.

## Data Availability

The authors do not have permission to share data.

## Appendix A Supplementary data

Supplementary Fig. 1 Prevalence ratios (95% confidence intervals) for undergoing wellness examinations among wives whose husbands underwent health checkups compared to wives whose husbands did not, using a nationally representative database from 2019 in Japan using multiple imputations by chained equations. *Note:* (1) Stratification was based on the wife's medical insurance type, including national health insurance, employee insurance (employee), and employee insurance (family); (2) Adjusted for place of residence; household expenditure; preschool children in the household; status of wife namely, age, education, smoking history, drinking habits, and subjective health perceptions; and K6 scores; (3) The participants were 51,874 couples aged 40–64, excluding 888 couples with missing medical insurance information.

Supplementary Fig. 2 Prevalence ratios (95% confidence intervals) for undergoing health checkups among husbands whose wives underwent health checkups compared to husbands whose wives did not, using a nationally representative database from 2019 in Japan. *Note:* (1) Stratification was based on the husband 's medical insurance type, including national health insurance, employee insurance (employee), and employee insurance (family); (2) Adjusted for place of residence; household expenditure; preschool children in the household; husband 's status namely, age, education, smoking history, drinking habits, and subjective health perceptions; and K6 scores; (3) The participants were 41,031 couples aged 40–64 with no missing data for the variables analyzed.

Supplementary Fig. 3 Prevalence ratios (95% confidence intervals) for undergoing wellness examinations among wives whose husbands underwent health checkups compared to wives whose husbands did not, using a nationally representative database from 2019 in Japan. *Note:* (1) Stratification was based on the wife's medical insurance type, including national health insurance, employee insurance (employee), and employee insurance (family); (2) Adjusted for place of residence; household expenditure; preschool children in the household; wife's and husband’s status namely, age, education, smoking history, drinking habits, and subjective health perceptions; and K6 score; (3) The participants were 39,935 couples in the health checkup analysis, 38,692 couples in the cervical cancer screening analysis, and 38,716 couples in the breast cancer screening analysis aged 40–64 with no missing data for the variables analyzed.

Supplementary Table 1 Multivariable modified Poisson regression analysis results with outcome as undergoing wellness examinations by wives, using a nationally representative database from 2019 in Japan. *Note:* *P-value < 0.05.
