# Supplementary material for: Relationship between health checkups and cancer screenings of wives and health checkups of their husbands: A cross-sectional study in Japan
Source: Prev Med Rep. 2024 Mar 23;41:102701. doi: 10.1016/j.pmedr.2024.102701 (PMC10987899; doi:10.1016/j.pmedr.2024.102701)
Supplement: Supplementary Fig. 1 — Prevalence ratios (95% confidence intervals) for undergoing wellness examinations among wives whose husbands underwent health checkups compared to wives whose husbands did not, using a nationally representative database from 2019 in Japan using multiple imputations by chained equations. Note: (1) Stratification was based on the wife's medical insurance type, including national health insurance, employee insurance (employee), and employee insurance (family); (2) Adjusted for place of residence; household expenditure; preschool children in the household; status of wife namely, age, education, smoking history, drinking habits, and subjective health perceptions; and K6 scores; (3) The participants were 51,874 couples aged 40–64, excluding 888 couples with missing medical insurance information. [file mmc1.pptx]

## Slide 1
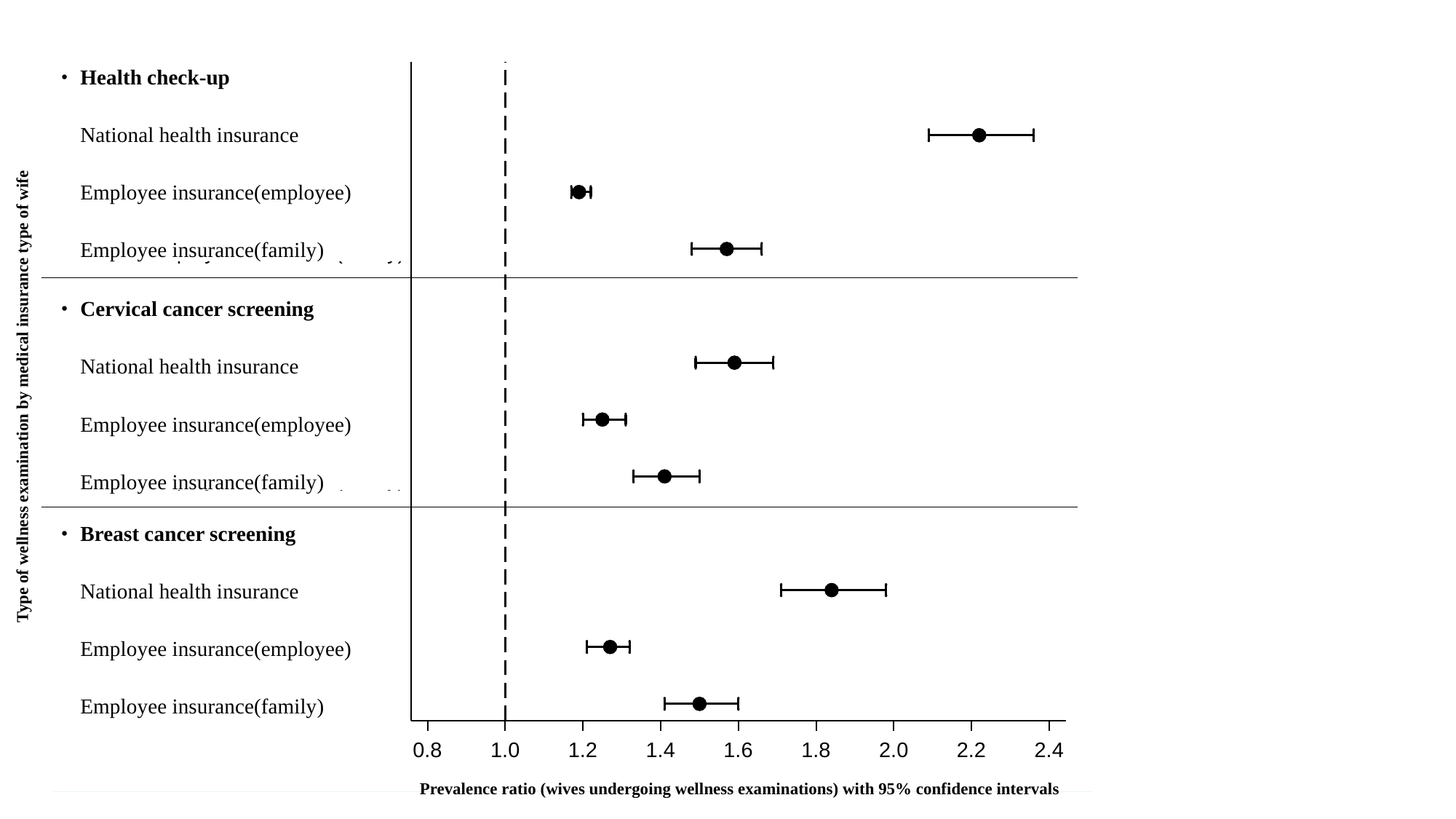

・Health check-up
　National health insurance
　Employee insurance(employee)
　Employee insurance(family)
Type of wellness examination by medical insurance type of wife
・Cervical cancer screening
　National health insurance
　Employee insurance(employee)
　Employee insurance(family)
・Breast cancer screening
　National health insurance
　Employee insurance(employee)
　Employee insurance(family)
Prevalence ratio (wives undergoing wellness examinations) with 95% confidence intervals
