# Supplementary material for: Relationship between health checkups and cancer screenings of wives and health checkups of their husbands: A cross-sectional study in Japan
Source: Prev Med Rep. 2024 Mar 23;41:102701. doi: 10.1016/j.pmedr.2024.102701 (PMC10987899; doi:10.1016/j.pmedr.2024.102701)
Supplement: Supplementary Fig. 2 — Prevalence ratios (95% confidence intervals) for undergoing health checkups among husbands whose wives underwent health checkups compared to husbands whose wives did not, using a nationally representative database from 2019 in Japan. Note: (1) Stratification was based on the husband 's medical insurance type, including national health insurance, employee insurance (employee), and employee insurance (family); (2) Adjusted for place of residence; household expenditure; preschool children in the household; husband 's status namely, age, education, smoking history, drinking habits, and subjective health perceptions; and K6 scores; (3) The participants were 41,031 couples aged 40–64 with no missing data for the variables analyzed. [file mmc2.pptx]

## Slide 1
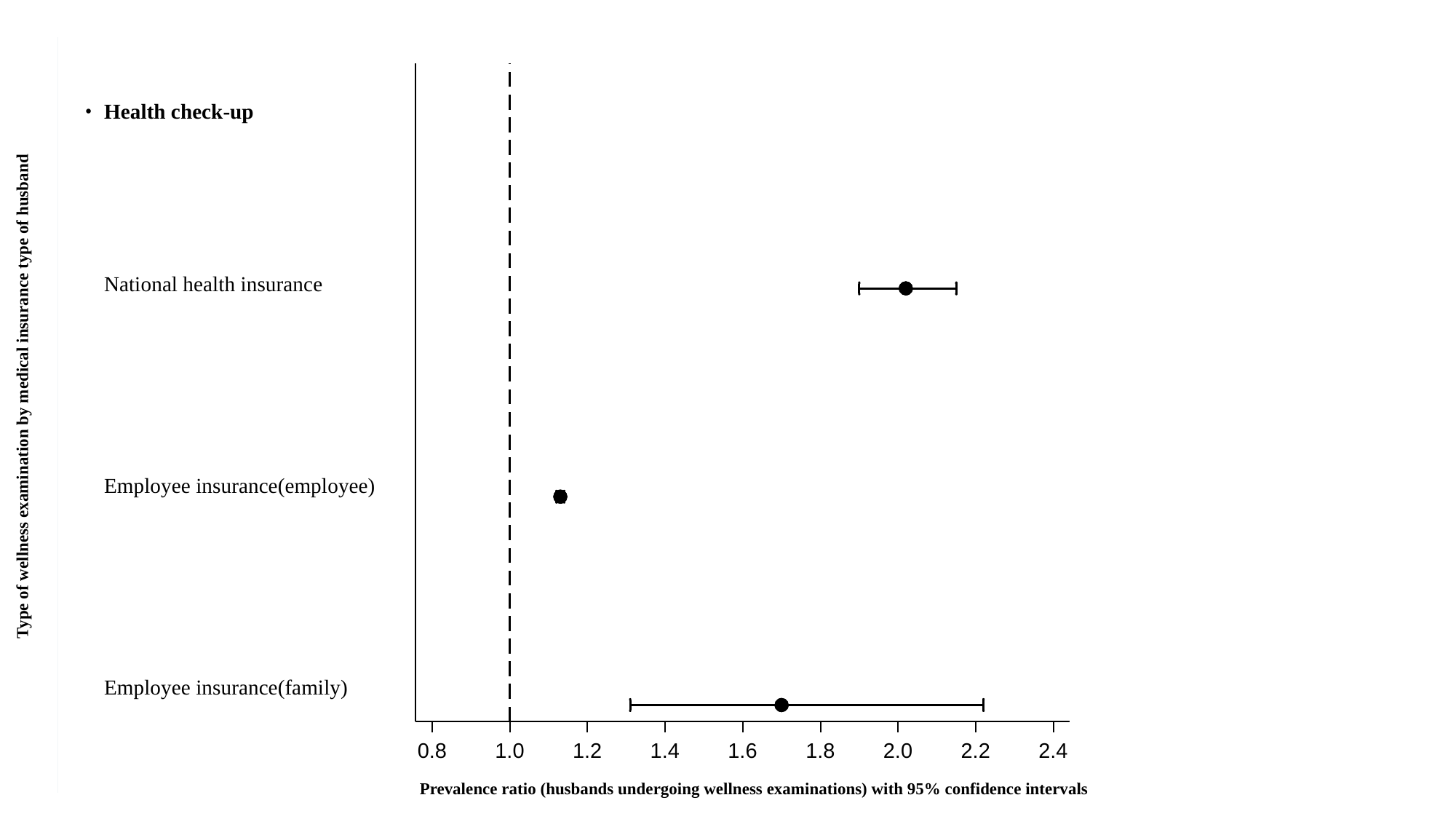

・Health check-up
　National health insurance
　Employee insurance(employee)
　Employee insurance(family)
Type of wellness examination by medical insurance type of husband
Prevalence ratio (husbands undergoing wellness examinations) with 95% confidence intervals
