# Supplementary material for: Relationship between health checkups and cancer screenings of wives and health checkups of their husbands: A cross-sectional study in Japan
Source: Prev Med Rep. 2024 Mar 23;41:102701. doi: 10.1016/j.pmedr.2024.102701 (PMC10987899; doi:10.1016/j.pmedr.2024.102701)
Supplement: Supplementary Fig. 3 — Prevalence ratios (95% confidence intervals) for undergoing wellness examinations among wives whose husbands underwent health checkups compared to wives whose husbands did not, using a nationally representative database from 2019 in Japan. Note: (1) Stratification was based on the wife's medical insurance type, including national health insurance, employee insurance (employee), and employee insurance (family); (2) Adjusted for place of residence; household expenditure; preschool children in the household; wife's and husband’s status namely, age, education, smoking history, drinking habits, and subjective health perceptions; and K6 score; (3) The participants were 39,935 couples in the health checkup analysis, 38,692 couples in the cervical cancer screening analysis, and 38,716 couples in the breast cancer screening analysis aged 40–64 with no missing data for the variables analyzed. [file mmc3.pptx]

## Slide 1
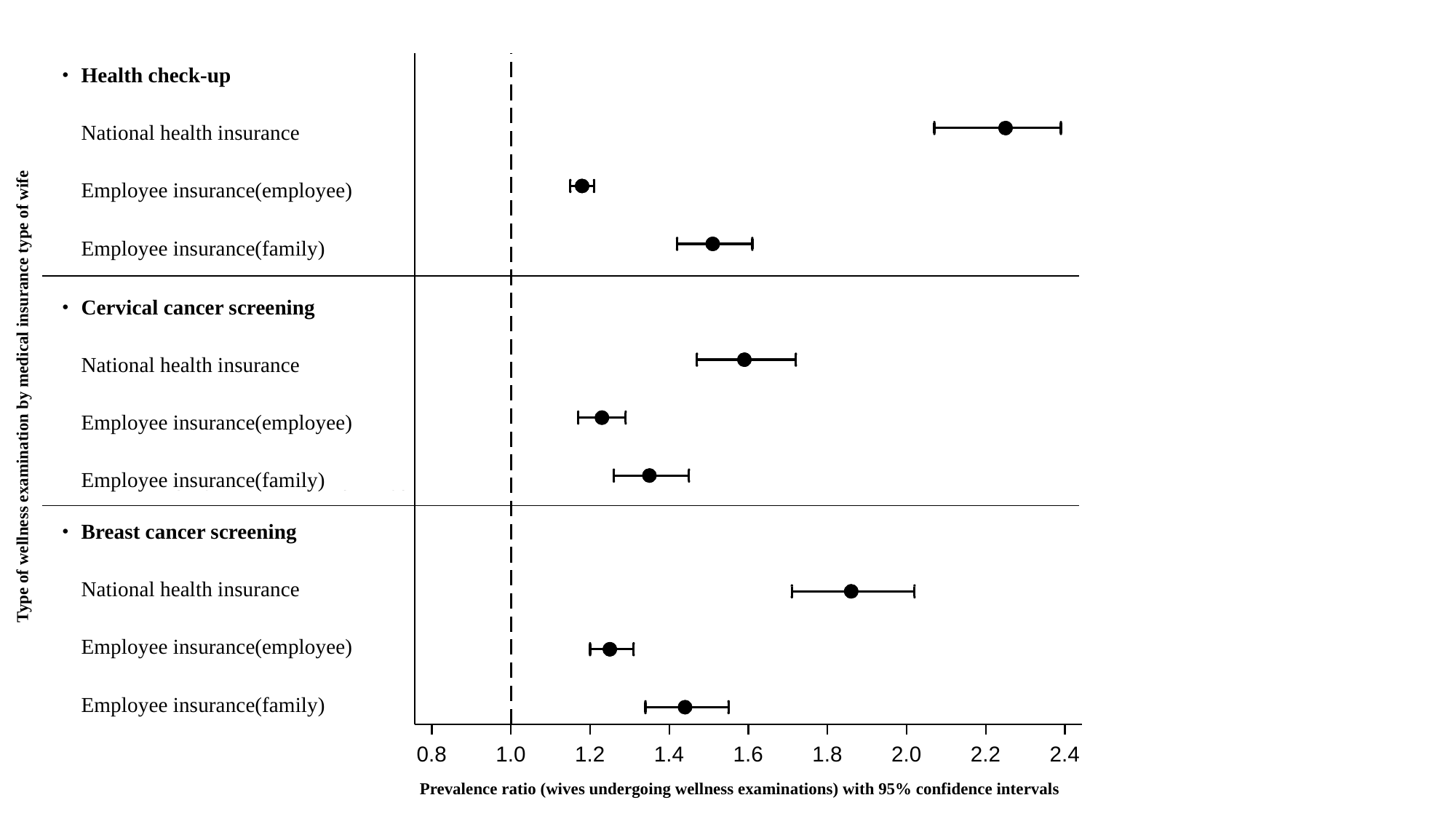

・Health check-up
　National health insurance
　Employee insurance(employee)
　Employee insurance(family)
Type of wellness examination by medical insurance type of wife
・Cervical cancer screening
　National health insurance
　Employee insurance(employee)
　Employee insurance(family)
・Breast cancer screening
　National health insurance
　Employee insurance(employee)
　Employee insurance(family)
Prevalence ratio (wives undergoing wellness examinations) with 95% confidence intervals
